# Toxic Epidermal Necrolysis Secondary to Post-surgical Analgesia for Posterior Vaginal Repair

**DOI:** 10.7759/cureus.113168

**Published:** 2026-07-22

**Authors:** Luis Mendoza-Razo, Jose Alberto Domínguez-López, Alejandra Jazmín Garibay-Partida, Manuel Guadalupe Díaz-Sánchez

**Affiliations:** 1 Intensive Care Unit, Instituto de Salud de Chiapas, Tuxtla Gutiérrez, MEX; 2 Genetics, Hospital Civil de Guadalajara "Fray Antonio Alcalde", Tuxtla Gutiérrez, MEX; 3 Dermatology, Hospital de Especialidades Centro Medico Nacional Siglo XXI, Mexico City, MEX; 4 General Medicine, Intercultural University of Chiapas, Tuxtla Gutiérrez, MEX

**Keywords:** critical care outcomes, drug-related adverse reactions, postoperative complications, stevens-johnson syndrome, toxic epidermal necrolysis

## Abstract

A 26-year-old woman with a history of epilepsy and drug hypersensitivity developed toxic epidermal necrolysis (TEN) following a posterior vaginal repair and levatoroplasty. She experienced fever, general malaise, and rapidly progressing blistering dermatosis, with areas of denuded epidermis and oral mucosal involvement. A dermatological assessment confirmed that her skin involvement exceeded 30%, leading to a clinical diagnosis of TEN. She was transferred to the intensive care unit, where treatment included discontinuation of the suspected drug, intravenous immunoglobulin, systemic corticosteroids, advanced pain management, fluid and electrolyte control, and sterile skin care. The patient showed no signs of sepsis, experienced progressive re-epithelialization, and demonstrated clinical improvement, allowing for discharge with specialized follow-up and strict avoidance of the implicated medications.

## Introduction

Toxic epidermal necrolysis (TEN) is a severe, immune-mediated, potentially life-threatening adverse skin reaction. It is characterized by massive necrosis of keratinocytes and epidermal detachment affecting more than 30% of the body surface area, along with extensive mucosal involvement. TEN is part of the Stevens-Johnson syndrome/TEN spectrum, distinguished by its greater skin involvement and poorer prognosis, with mortality rates ranging from 25% to 50%, depending on clinical severity and patient comorbidities [1].

Pathophysiologically, TEN is associated with a cytotoxic response mediated by CD8+ T lymphocytes, the release of granulysin, perforins, and granzymes, as well as the activation of apoptotic and necroptotic pathways leading to widespread epidermal destruction.

The most common cause of TEN is drug exposure, with antibiotics, non-steroidal anti-inflammatory drugs (NSAIDs), aromatic anticonvulsants, and allopurinol being some of the main agents involved. In a hospital setting, particularly in the postoperative period, the risk increases due to the simultaneous use of multiple drugs, analgesics, prophylactic antibiotics, and anti-inflammatories, which can act as triggers in susceptible individuals. This environment complicates early identification of the causal agent and may delay the timely discontinuation of the responsible drug, thereby allowing progression of skin injury [2].

Clinically, TEN often begins with nonspecific prodromal symptoms such as fever, general malaise, sore throat, and flu-like symptoms, followed by the appearance of painful erythematous macules that converge, evolve into blisters, and culminate in epidermal detachment, with a positive Nikolsky sign. Mucosal involvement (oral, ocular, and genital) is common and significantly contributes to morbidity. The extensive loss of skin barrier results in a pathophysiological condition comparable to that of severe burns, with fluid and electrolyte imbalances, a high risk of secondary infections, multi-organ dysfunction, and intense pain [3].

Management of TEN requires a multidisciplinary approach and, in most cases, treatment in an intensive care unit or specialized burn unit. Key measures include the immediate discontinuation of the suspected drug, hemodynamic support, strict fluid and electrolyte management, advanced pain control, meticulous skin care, and infection prevention. In certain cases, immunomodulatory therapies such as intravenous immunoglobulin, systemic corticosteroids, or biological agents may be considered, although their use remains a topic of debate [4].

We present the case of a young woman who required management in the intensive care unit.

## Case presentation

A 26-year-old woman had a history of childhood-onset epilepsy and had previously been treated with carbamazepine. However, the medication was discontinued due to a generalized itchy rash. She also reported additional medication hypersensitivities, including intolerance to amoxicillin, clindamycin, and vitamin B complex.

The patient was scheduled for elective surgical correction of a vaginal septum by the gynecology department. In the immediate postoperative period, she received 30 mg of intravenous ketorolac. Following the first dose, she developed malaise, pain, and pruritus, followed by the appearance of erythema and epidermal detachment involving the scalp, trunk, and lower extremities.

Initially, the lesions were characterized by polymorphic purpuric erythematous macules with dark centers and a target-like morphology of varying sizes, with a tendency to coalesce into plaques involving approximately 20% of the body surface area. The lesions rapidly progressed to flaccid bullae and extensive areas of denuded epidermis with a scalded-skin appearance and a positive Nikolsky sign. Ultimately, skin involvement extended to 45% of the total body surface area, as estimated using the Wallace rule of nines.

**Figure 1:**
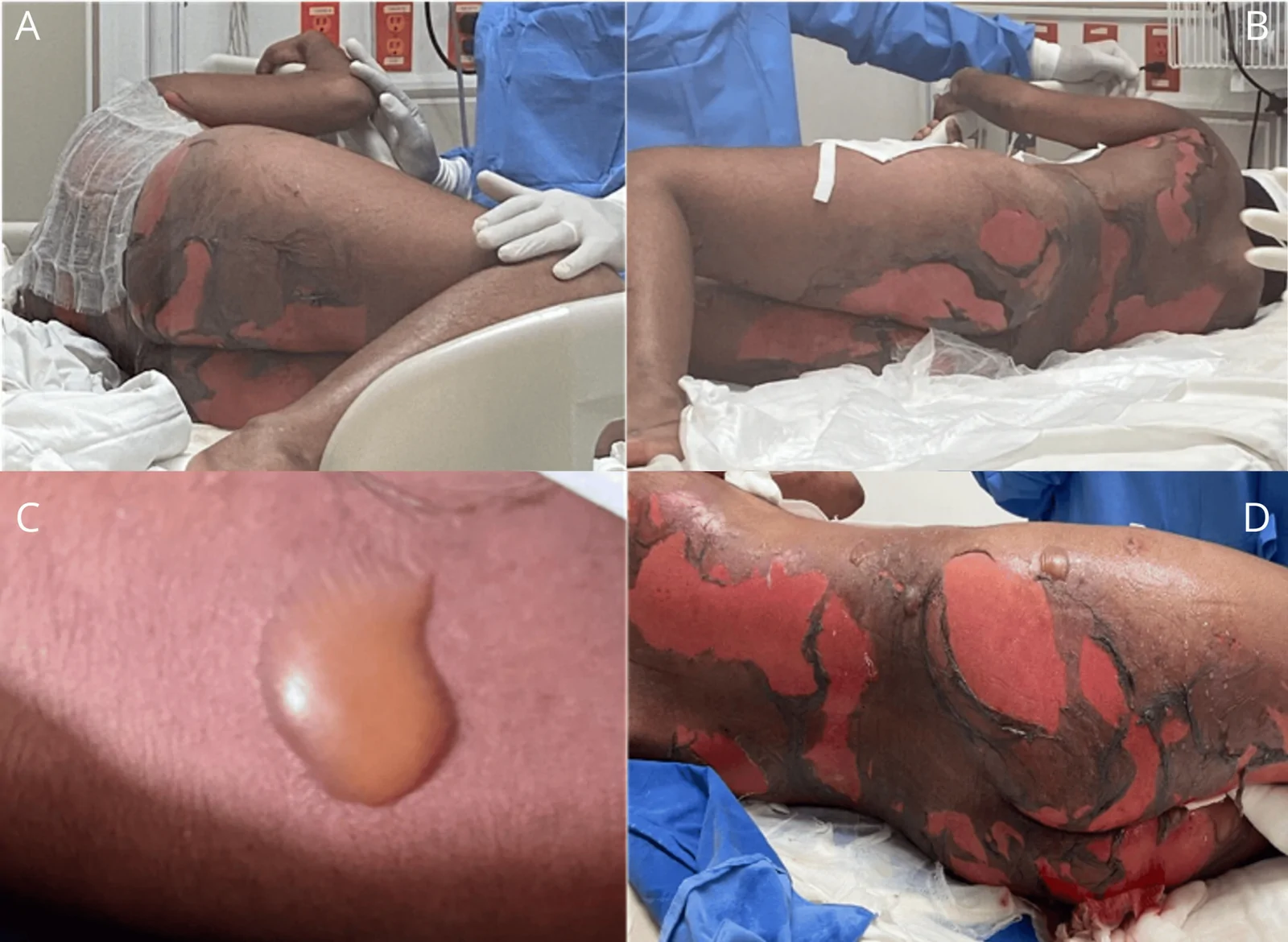
Cutaneous involvement during the patient's stay in the intensive care unit A large proportion of the body surface area was affected. The presence of bullous lesions and epidermal detachment was consistent with a positive Nikolsky sign, defined as epidermal separation induced by lateral pressure on clinically unaffected skin due to epidermal necrosis.

Mucosal involvement was also documented. Oral lesions affected the upper and lower lips as well as the buccal mucosa and were characterized by whitish pseudomembranes surrounded by an erythematous halo. Genital involvement included the labia majora, labia minora, and perianal region, presenting as superficial erosions. A Severity-of-Illness Score for Toxic Epidermal Necrolysis (SCORTEN) score of 2 was calculated (Figure 1).

A clinical diagnosis of TEN was established. Skin biopsy was not performed because the clinical presentation was highly characteristic, and the primary focus of this report extended beyond the dermatological aspects of the disease. Treatment was initiated with intravenous immunoglobulin at a dose of 0.2 g/kg/day for three consecutive days, and the patient was transferred to the intensive care unit for specialized management.

**Figure 2:**
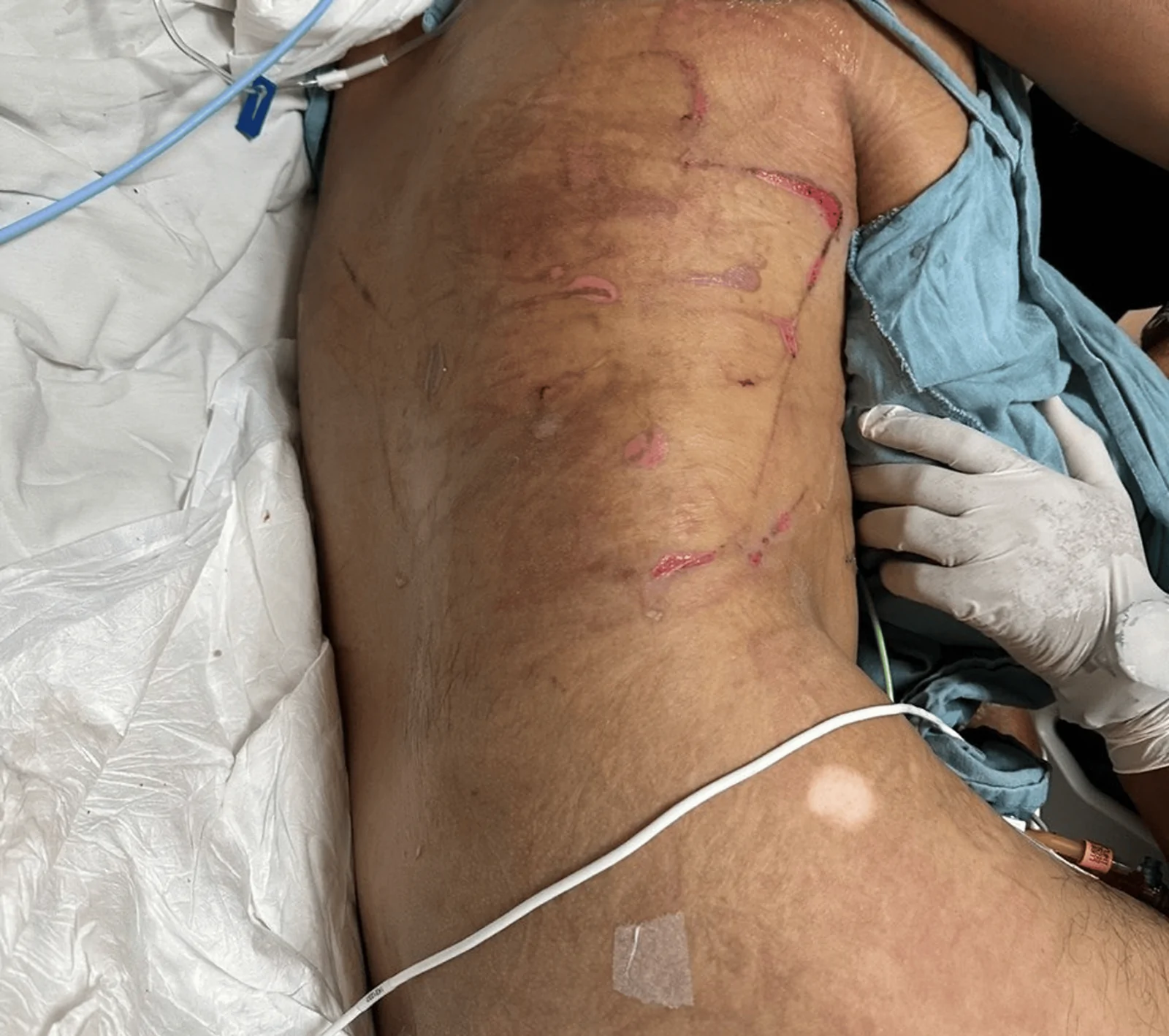
Follow-up of the skin lesions at the internal medicine department

Cutaneous care consisted of sterile dressings applied twice daily and regular use of emollients. Over the following days, the skin lesions showed gradual re-epithelialization without the development of new areas of epidermal detachment (Figure 2).

Her subsequent recovery was satisfactory, with no recurrence of skin lesions at the initial outpatient follow-up. She was discharged from the hospital with follow-up by dermatology and allergy and strict instructions to avoid the suspected drugs.

## Discussion

Stevens-Johnson syndrome and TEN are part of a spectrum of severe mucocutaneous reactions characterized by extensive epidermal necrosis and skin detachment. The distinction between these two conditions is primarily based on the percentage of body surface area affected, with TEN diagnosed when involvement exceeds 30%. This condition is associated with a morbidity and mortality rate that can reach up to 48% in the most severe cases. Therefore, early diagnosis, proper classification, and identification of clinical risk factors are crucial for prognosis [4,5].

Clinically, the initial phase is often nonspecific, with systemic prodromal symptoms such as fever, general malaise, sore throat, and upper respiratory symptoms, followed by the appearance of erythematous macules or confluent lesions mainly on the trunk, which progress centrifugally. Blisters and areas of detached epidermis then develop, which can be elicited with minimal lateral pressure on the skin, a manifestation known as the Nikolsky sign, a key clinical finding in diagnosing the condition [6,7].

Although TEN is a rare condition, it is essential to emphasize the history of drug exposure, as most cases are mediated by drug-induced immunological mechanisms. Individual susceptibility has been associated with genetic factors, especially certain HLA alleles and the major histocompatibility complex. However, infectious triggers, such as *Mycoplasma pneumoniae* and herpes simplex virus, have also been described. The most implicated drugs include antibiotics, non-steroidal anti-inflammatory drugs, and antiepileptics. In the presented case, a history of previous reactions to anticonvulsants suggests an immunological predisposition to severe drug-induced skin reactions [8,9].

The SCORTEN scale is used for prognostic stratification, incorporating variables such as age, heart rate, presence of malignancy or comorbidities, percentage of epidermal detachment, and serum levels of urea, glucose, and bicarbonate, allowing estimation of mortality risk. The pathophysiology involves a cytotoxic immune response primarily mediated by CD8+ T lymphocytes, which induce massive apoptosis of keratinocytes through interaction with antigens presented on the cell surface. Recent studies have identified associations between specific HLA alleles and adverse reactions to certain drug groups [9,10].

Additionally, monocytes and other immune system cells participate in amplifying the inflammatory cascade, contributing to cell death through mechanisms such as necroptosis. In the diagnostic approach, a detailed medical history with an emphasis on recent drugs is essential. When a clear pharmacological trigger is not identified, infectious etiologies should be considered. Recommended laboratory studies include a complete blood count, erythrocyte sedimentation rate, and C-reactive protein; in selected contexts, expanding the diagnostic workup to include immunological or rheumatological profiles may help rule out differential diagnoses [7,8].

In the described patient, the acute onset of extensive skin lesions following postoperative drug administration, along with mucosal involvement and epidermal detachment greater than 30%, supported the clinical diagnosis of TEN. Clinical practice guidelines recommend management in intensive care units or areas experienced in treating burn patients, immediate discontinuation of suspected drugs, strict fluid and electrolyte control, close monitoring for complications, and implementation of local skin care with sterile dressings. The use of systemic corticosteroids should be evaluated individually, considering the risk-benefit ratio, while comprehensive support and prevention of infectious complications are the cornerstones of treatment [9,10].

## Conclusions

Although the development of TEN cannot be entirely prevented, obtaining a thorough medical history, including any previous drug hypersensitivity reactions, is essential to minimize the risk of severe adverse drug events. This case highlights the importance of early recognition of drug-induced reactions and the prompt discontinuation of the offending agent. Furthermore, the patient's favorable outcome, despite the severity of the condition, underscores the critical role of timely specialized management and multidisciplinary care within the intensive care unit, which may significantly improve outcomes in patients with TEN.
